# Challenging case of treating fungal keratitis

**DOI:** 10.22336/rjo.2022.14

**Published:** 2022

**Authors:** Darko Ler, Aida Pidro, Ajla Pidro Miokovic

**Affiliations:** * Ophthalmology Department, “Prim. Dr. Abdulah Nakas” General Hospital, Sarajevo, Bosnia and Herzegovina; ** Ophthalmology Policlinic Anda, Zagreb, Croatia

**Keywords:** fungal keratitis, intrastromal application, antifungal medication

## Abstract

**Objective:** Treating fungal keratitis is always a challenge due to limited antifungal medication, their poor penetration, and often, the delay in diagnosis. The purpose of this study is to present new routes of medication delivery, such as intrastromal application for advanced cases not responding to topical therapy.

**Methods:** A 60-year-old female presented with fungal keratitis, complaining of decreased vision, redness in her left eye followed by frequent pain, which got worse a few days prior. Visual acuity was limited to hand motion. Slit-lamp examination showed advanced conjunctival hyperemia, a very deep white feathery lesion in the central cornea, with satellite lesions on the periphery, and pseudohypopyon of 3 mm in the anterior chamber. She was started on oral fluconazole 100 mg b.i.d, followed by corneal debridement and topical hourly voriconazole eye drops and intrastromal voriconazole application as her local findings did not show any improvement.

**Results:** After repeating the procedure three times, the inflammatory reaction was decreased, the central ulcer healed, satellite lesions disappeared and pseudohypopyon did not recur even after discontinuation of the topical therapy.

**Conclusion:** Intrastromal voriconazole application showed its safety and efficacy in treating advanced fungal keratitis with deep intrastromal lesions.

**Abbreviations:** NSAID = non-steroid anti-inflammatory drugs, BCVA = best corrected visual acuity, MIC = mean inhibitory concentration

## Introduction

Fungal keratitis is an infectious corneal disease that often has worse prognosis compared to other causes of keratitis with different risk factors contributing to fungal infection, such as prolonged or inadequate use of corticosteroid topical therapy, topical antibiotics, prolonged contact lens usage, ocular surface disease, or vegetative trauma [**1**].

There are many limitations to treating fungal keratitis, which include delayed diagnosis, limited spectrum, and availability of both systemic and topical antimycotic, poor drug penetration, delayed healing, toxicity, recurrence, thinning of the cornea, and possibility for perforation [**2**]. If there is no response to the medication, a surgical approach is often needed and includes epithelial debridement, therapeutic keratectomy, or keratoplasty. Limitations to keratoplasty are high risk of transplant rejection, high cost of the surgery, and unavailability of donor tissue [**3**]. Considering that, the different surgical approach should be used to decrease the inflammation, induce stromal healing, and prevent perforation, and decrease scar formation. 

This case report aims to present a treatment protocol used for the treatment of a severe case of deep stromal fungal keratitis, which included surgical corneal epithelial debridement with superficial keratectomy and intrastromal antimycotic injection.

## Methods

We describe a case of a 60-year-old female patient who came to our department complaining of decreased vision and slight pain in her left eye, with “white spots on her eye” that appeared a few days prior. Her medical history revealed that she has struggled with treating her left eye over the last 12 months, visiting different ophthalmologists. Her main complaints were constant tearing and redness in her left eye. She denied using contact lenses or systemic immunosuppressive therapy. She reported that the possible injury to her eye was with a paper tissue that she used to remove the excess tears. She denied other vegetative injuries. There was no history of another systemic disease.

Past medical records showed the first diagnosis of superficial herpes simplex keratitis that was properly treated with topical antivirotic and antibiotic therapy. On her next visit one week after, she was prescribed topical corticosteroids by another ophthalmologist. She had over 20 visits to the ophthalmologist complaining of the same symptoms and she was on-and-of topical non-steroid anti-inflammatory drugs (NSAIDs) and corticosteroids combined with different artificial tears and gels. All that time, her right eye was healthy without any symptoms.

**Fig. 1:**
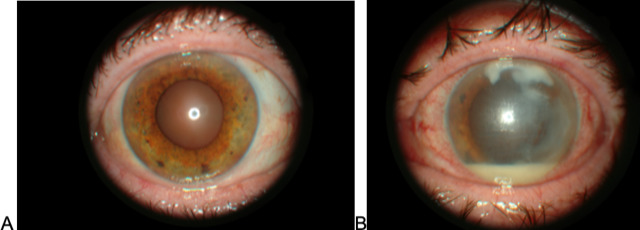
Slit-lamp examination on the first visit, **A** - right eye, **B** - left eye

The best corrected visual acuity (BCVA) on her right eye was 0.8. The anterior segment was normal with incipient lens opacities (**Fig. 1A**). BCVA on her left eye was limited to hand motion. Slit-lamp examination revealed mixed conjunctival hyperemia, uneven and bumpy corneal surface with decreased transparency, and very deep white feathery lesion in the central cornea with satellite lesions on the periphery, with the largest lesion on the superior cornea. The anterior chamber had a pseudohypopyon of 2.5 mm (**Fig. 1B**). The lens and the fundus were not visible during the examination. B scan was normal. We took a conjunctival swab and performed corneal scraping using 15-blade under topical anesthesia that was then all sent to the microbiology for fungal testing, which came positive after 14 days and revealed the presence of Penicillium spp. 

The first problem in treating fungal keratitis was the unavailability of topical antifungal medication. The patient was started on oral fluconazole 100 mg b.i.d., with topical moxifloxacin and chloramphenicol due to their positive and synergistic effect on fungal keratitis [**4**]. As we have managed to get to voriconazole, different concentrations for topical, intracameral, and intrastromal applications were made. 

Voriconazole (200 mg) for parenteral use was diluted firstly to the concentration of 1% (10 mg/ ml) to be used as topical medication every hour for 2 weeks. The solution was stored for 7 days at a temperature of 2-8°C in aseptic conditions [**5**]. Afterward, 1 ml of this solution was diluted with 19 ml of ringer lactate to get a final concentration of 50 µg/ 0.1 ml for intrastromal application [**5**]. The solution was prepared fresh in the same way each time.

**Fig. 2:**
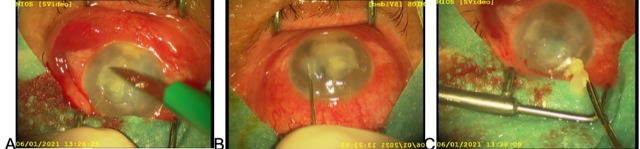
The first surgery, **A** - corneal debridement, **B** - intrastromal voriconazole application, **C** - pseudohypopyon evacuation

**Fig. 3:**
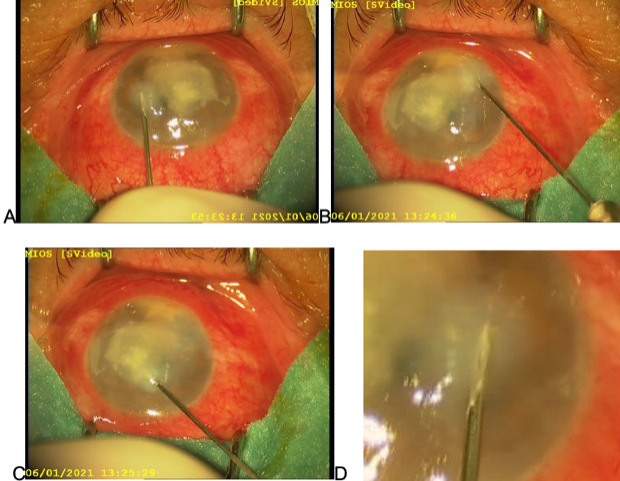
Intrastromal application technique, **A-C** - several injection sites surrounding the lesion circularly, forming a barrage around the ulcer area, **D** - hydration of injection site

The procedure was performed in the operation theater. After preparing the surgical field and draping the patient, under topical anesthesia with 0.5% tetracaine, corneal debridement was performed to remove all the necrotic tissue and to improve topical drug penetration [**6**]. It was done using a 15-blade removing layer by layer (**Fig. 2A**). Since the lesion was very deep, it was not possible to remove the infiltrate totally without perforating the cornea. After the debridement, a 27-gauge needle was inserted under an oblique angle and with the bevel turned down in the area of the clear cornea, around the affected area (**Fig. 2B**). Around 0.1 ml of voriconazole was inserted into several injection sites around the lesion in 4-6 divided doses surrounding the lesion circularly, forming a barrage around the ulcer area (**Fig. 3A-C**). The drug was injected until the injection site was hydrated due to swelling in the middle and deep areas of the cornea, which is a sign of drug penetration and deposit (**Fig. 3D**). Paracentesis was afterward performed, pseudohypopyon evacuated, followed by intracameral injection of voriconazole (**Fig. 2C**). 

## Results

**Fig. 4:**
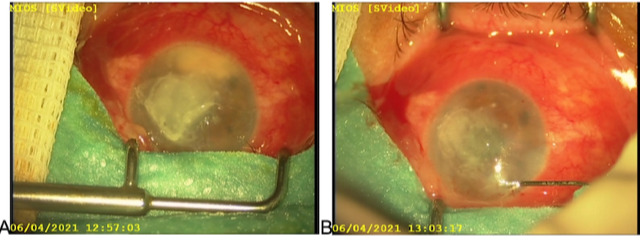
Anterior segment findings, **A** - prior second surgical procedure, **B** - after second surgical procedure (notice barrage white dots on the cornea, indicating previous injection sites and drug deposits)

Our patient was examined daily and the progression was documented along with the assessment of repeating the injections. There was a small improvement the day after but it was followed by pseudohypopyon recurrence (**Fig. 4**). The procedure was repeated three times in the interval of 5 days between the procedures. Autologous serum was injected a few times subconjunctivally to improve corneal healing. 

As we managed to get hold of topical natamycin, we started it hourly instead of topical voriconazole. Corneal scraping revealed Penicillium spp. Penicillium is filamentous fungi. Systemic fluconazole was discontinued from the therapy since it is more effective for yeast than for filamentous fungi. Our patient’s therapy remained topical natamycin for 6 weeks, with a taper as the local findings were improved. After the cornea has completely healed, a low dosage of topical steroids was introduced and, as the patient showed significant improvement in a form of decreased inflammation, the dosage was increased to 4 times a day with a gradual taper over one month period, which helped significantly in the reduction of corneal scarring. 

**Fig. 5:**
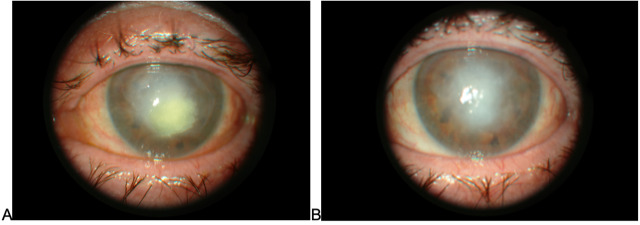
Anterior segment examination after discontinuation of all medication, **A** - one month without therapy, **B** - three months without therapy

Our patient showed a good response for this combined therapy and is stable for three months already without therapy, without recurrence, as the medication was withdrawn (**Fig. 5A, B**). The improvement was defined as decreased inflammation, reepithelization, and healed corneal ulcer, the disappearance of satellite lesions and pseudohypopyon, decrease in central corneal opacity, reduction in the size and transparency of the scar, and the patients’ subjective improvement. The visual acuity did not improve due to the localization of the remaining corneal scarring. The next step would be corneal transplantation to regain her visual function, which the patient has decided to postpone for now.

## Discussion

There are many challenges in treating advanced fungal keratitis. The limitation of topical antifungal medications is their poor penetration due to their high molecular size and toxicity [**7**] that leads to suboptimal results of the treatment. Many studies proved the efficacy of combined topical and systemic antifungal medications in the early stages of keratitis [**8**]. This led to the need of finding alternative methods of reaching the fungi with the intracameral and intrastromal application. Intracameral application has its limitations due to the rapid decrease in concentration in aqueous humor after injecting the medication, while intrastromal injection maintains effective high concentration for about 7 days [**7**]. The intrastromal injection is thus a targeted drug delivery to infectious deep stromal areas, which can be used in fungal keratitis that does not respond to topical therapy. 

Voriconazole has shown better stromal penetration compared to other antifungal topical medication, but its penetration was still described as limited, especially for deeper lesions [**9**]. Some studies are describing intrastromal antifungal medication delivery to increase its concentration in deep stromal lesions [**10**]. Even though the concentration of medicine was increased, there is not enough evidence that this type of treatment is efficient in fungal keratitis [**10**]. Natamycin is the only commercially available antifungal medication and is shown to be effective against both yeast and filamentous fungi. Voriconazole has also been shown to be effective against broad-spectrum fungus including Fusarium and Aspergillus [**11**]. One of the other antifungal medications is amphotericin B, which can be used in natamycin-resistant cases in treating filamentous fungi, and has poor penetration and high toxicity [**7**]. Even though it can be applied intrastromal, some studies are describing more complications in a form of toxicity of a corneal surface as well as renal toxicity [**12**]. Voriconazole showed fewer adverse effects and better results due to better penetration and lower mean inhibitory concentration (MIC) [**2**]. It is important to perform corneal debridement frequently to remove necrotic tissue and to allow better drug penetration. 

Many studies report that it is usually required to repeat intrastromal injections at least two to three times for the treatment to be effective [**13**]. Maniam et al. also reported an improvement after two to three injections [**8**]. Kalaiselvi et al. reported the improvement in 72% of patients treated with more than one injection of intrastromal voriconazole that was previously treated and did not respond to topical medication [**10**]. Another study showed improvement in 14 out of 20 patients, which required 2.65 injections on average [**14**]. It is important to mention that no adverse effects of intrastromal voriconazole application in a form of corneal haze, edema, erosions, and toxicity, which was consistent with cases from other studies [**8**].

All the studies mentioned reported an increase in the concentration of voriconazole, but its therapeutic effect is still under investigation and more studies with a higher number of patients will have to be performed. The reasons for that could be the ununiformed drug delivery due to variable concentration or distribution around the lesion [**15**]. Advanced prospective randomized multicentric studies with a large sample size could help in forming the uniform algorithm in the intrastromal application that would help validate the safety and efficacy of the procedure.

## Conclusion

Corneal debridement is a necessary way to remove necrotic tissue and to allow the medication to reach the infection site. Intrastromal medication injection provides its direct delivery, improves penetration, allows the medication to be deposited in the stroma slowly releasing it, and decreases the toxicity of the medication. The intrastromal antifungal injection is a targeted way of delivering antifungal medication in the appropriate concentration and should be used in deep fungal ulcers that do not respond to topical medication.

**Conflicts of Interest statement**

The authors declare that there are no conflicts of interest. 

**Informed Consent and Human and Animal Rights statement**

Informed consent has been obtained from a patient included in this study. 

**Authorization for the use of human subject**

Ethical approval: The case report related to human use complies with all the relevant national regulations, institutional policies, is in accordance with the tenets of the Helsinki Declaration, and has been approved by the review board of the Veneto Eye Bank Foundation, Venice, Italy. 

**Acknowledgments**

None.

**Sources of Funding**

None.

**Financial Disclosure(s)**

None.
